# Supplementary material for: Are carbon emissions trading and green financial instruments synergistic? -Comprehensive quantitative research based on content analysis
Source: PLoS One. 2024 Mar 7;19(3):e0298601. doi: 10.1371/journal.pone.0298601 (PMC10919601; doi:10.1371/journal.pone.0298601)
Supplement: S1 Data — (ZIP) [file pone.0298601.s001.zip › Data/地级市Z_F工作报告.zip!/╔┬╬≈/░▓┐╡╩╨/└┤╫╘╣1⁄2╓┌║┼í╛┬φ┐╦╩2╛▌═°í┐_2013.docx]

各位代表：

　　现在，我代表市人民政府向大会作工作报告，请予审议。并请各位政协委员和列席会议的同志提出意见。

　　一、2012年工作回顾

　　过去的一年，我们在市委的正确领导下，坚持以科学发展观为指导，紧紧围绕循环发展、富民强市主题，不断夯实推进措施和发展基础，经过全市人民的共同奋斗，经济平稳较快发展，社会保持和谐稳定，全面完成市三届人大二次会议确定的各项任务，实现美好安康建设的良好开局。

　　全年实现生产总值513.02亿元，增长15.2%；规模以上工业增加值180.57亿元，增长33.9%；全社会固定资产投资380.27亿元，增长24.9%；财政总收入51.8亿元，增长19.1%，其中地方一般预算收入21.66亿元，增长25.2%；城镇居民人均可支配收入和农民人均纯收入分别达到20300元和5815元，增长16.9%和16.1%。单位生产总值能耗下降3.3%，汉江出境断面水质保持国家II类标准。经济发展的质量效益逐步提高，协调性、可持续性进一步增强。

　　（一）转变发展方式，循环产业体系建设取得新成效。新型工业快速增长。工业实现利税47.9亿元，增长53%，完成投资109.5亿元，增长76.5%。工业化率达到38%。新增规模工业企业74户、产值过亿元企业64户。白河专用车和商用车、旬阳球磨铸件、汉滨农地膜、汉阴富硒食品、岚皋复合材料等35个重点项目竣工投产。以汽车装配为主体的装备制造业实现产值30.3亿元，增长1.1倍，成为拉动工业增长新的生力军。先后制定出台扶持发展劳动密集型产业、大力发展山林经济和涉水产业的三个意见，有力促进了实体经济的发展。现代农业特色鲜明。加快实施“百园建设、千元增收”工程，启动建设现代农业园区154个，其中省级20个，建成面积21.4万亩，带动园区农民户均增收2686元。粮油总产98.7万吨，连续九年增产增收。肉类总产26.1万吨。新增设施蔬菜2.1万亩，发展水面养殖21.6万亩，种植烤烟22万亩。新建特色经济林43.5万亩，发展林下种植37.9万亩。汉滨区被列为国家现代农业示范区。紫阳富硒茶成为我市首个中国驰名商标。与中国农科院共建的中国（安康）富硒产品研发中心加快推进。生态旅游蓬勃发展。岚皋县创建为第一批省级旅游示范县。瀛湖创建为国家4A级景区。安康成为央视气象公共服务播报城市。第十二届中国安康汉江龙舟节、第八届中国盆景展、首届南宫山旅游文化节等重大节庆活动成功举办，安康生态旅游知名度明显提高。全年接待游客1830万人次，增长17.2%。积极推进三产服务业发展，新增限额以上商贸企业48户，全社会消费品零售总额151.3亿元，增长17%。园区经济活力增强。高新区生产总值增长30%以上，财政收入增长62.5%，新增亿元以上企业7户。县域工业园区入园企业达到624户，实现产值204亿元。

　　（二）突出项目建设，发展基础条件得到新改善。完善项目建设五项制度，有力推动重大项目建设。全市在建项目1415个，新开工项目885个，投资亿元以上的项目130个。投资对经济增长贡献率达到60%以上。新增项目建设用地1.1万亩。在中省投资大幅下降的情况下，地方投资快速增长，民间投资175亿元，增长45.9%，占固定资产投资的比重超过50%。十大建设项目扎实推进。全市人民关注的安康火电厂、安康新机场已经国家批准，东坝防洪保安工程加快建设，江北和9个县城污水处理厂、10县区垃圾无害化填埋场建成。西坝片区旧城改造启动征迁，东坝综合开发完成控制性规划和国际小商品城项目规划，城区北环线项目完成规划和可研工作，白河、石泉县城防洪工程开工建设，金属镍产业园和配套的白河硫精砂项目加快推进。十天高速安康至陕鄂界、安岚二级公路全线通车，安平高速控制性工程、西康铁路二线和紫阳两座汉江大桥加快建设，渔紫路和镇坪县城过境段等改造完成，全面取消政府还贷二级公路收费。建成通村示范路3500公里、连户路3100公里。水利建设完成投资13.8亿元，增长67%，汉江综合治理和丹治二期工程全面启动，引汉济渭工程进度加快，加固病险水库15座，治理中小河流50公里，解决31.8万人的安全饮水问题。汉中至安康天然气主管网及江北配套管网、3个110千伏输变电工程、市医疗废物处理中心、19个规模化畜禽养殖污染治理项目建成，淘汰黄标车2012辆，全年造林绿化66.4万亩。城市空气质量优良天数359天，环境风险防控和生态创建工作进一步加强。

　　（三）加快人口聚集，城镇化建设迈出新步伐。编制完成安康市和10县区城乡一体化发展规划，中心城市控规覆盖率100%。中心城市完成市政建设投资17.6亿元，增长44.3%。市委党校、安康学院、市法院等10个单位北迁，公安指挥中心、国土信息大厦等7个北迁工程加快实施。南环干道全线贯通，一江两岸亮化一期工程、内环路文化墙、水西门城楼、秦岭大道、汉江路等项目建成投用，新建6个市场、18处环卫设施。滨江三期、高新大道、高新创业大厦、高新中学等重点项目加快实施。中心城市绿化覆盖率达到38.6%，创建国家园林城市和国家卫生城市通过省级考核鉴定，实现省级园林县城全覆盖。启动数字安康建设。中心城区拆除违章建筑113户，清理违法用地和闲置土地8862亩。2011年避灾扶贫搬迁工程通过省政府验收，2012年实施避灾扶贫搬迁建房3.6万户，主体竣工3.1万户，集中安置率85%。恒口示范区和高新区两个跨区域集中安置示范小区开工建设。当年新增城镇个体工商户16879户、小微企业1100户、劳动密集型企业25户。8.68万农村居民进城入镇，全市城镇化率提高到39.4%。

　　（四）推进改革开放，经济发展活力有了新增强。我市被批准为全国发展改革试点城市。设立恒口统筹城乡示范区和瀛湖生态旅游区。制定出台金融支持实体经济、服务县域经济发展的意见，全市金融机构贷款余额308.97亿元，增长19.5%；全市财政设立5亿元的工业、农业、林业发展基金，扶持发展80个重点工业项目和150户劳动密集型企业。成立安康市发展投资集团有限公司，净资产达到43亿元，首期发行10亿元企业债券已报国家发改委待批。市财信担保公司资本金扩大到3.45亿元。新增小额贷款公司5家。新增国有商业银行县级支行1家。新增保障性住房等开发性贷款12亿元。全面实施32个县级公立医院综合改革，县区基层医疗卫生机构实现药品“三统一”和零差率销售，基本实现城乡公共卫生服务均等化。不断深化事业单位分类改革，精简各类事业机构507个。文化体制改革不断完善。公共资产管理改革取得阶段性成效。全面推行国库集中支付和公务卡制度，非税收入全部纳入综合预算管理。开展北京、江苏、福建、广东、港澳和西洽会、厦洽会大型招商活动，签订招商引资合同项目182个，其中投资10亿元以上6个，开工项目180个，到位资金208亿元。郭氏集团、紫金矿业、三一重工、帝奥电梯等21户中国500强和行业知名企业落户安康。

　　（五）加大民生投入，各项社会事业实现新进步。全市民生工程投入68.4亿元。平利“双高双普”正式验收命名，宁陕教育强县和白河县“双高双普”通过评估。石泉、岚皋实行免费高中教育，高中阶段入学率达到86.8%，高考二本上线人数8362人，文理科一本、二本上线率均居全省第二。建成45所公办幼儿园，学前教育三年净入园率达到75.9%。义务教育营养餐工程覆盖26万学生，资助家庭困难学生15.4万人次。建设保障性住房28598套，竣工25066套，完成投资67.2亿元，进度居全省第二。增加学位2000多个，增加病床位1300多张，开放13处人防地下停车场，中心城区上学难、住院难、行车难问题有所缓解。新增城镇就业1.58万人、大学生就业3640人、小额创业担保贷款11亿元，城镇登记失业率控制在3.25%。社会保障制度实现全覆盖，未参保的7586名集体企业超龄人员、“五七工”、“家属工”、宗教教职人员纳入基本养老保障统筹，企业退休人员养老金、企业军转干部生活补贴提标发放到位，城乡低保、五保户供养、困难救助等政策全面落实。农村五保集中供养率达到52%。新一轮扶贫开发扎实起步，全年脱贫10.4万人。市中医院门诊综合楼、市人民医院医技大楼竣工投用，市儿童医院开工建设，实施78个县镇社区卫生服务项目。计生奖扶政策全面落实，低生育水平保持稳定。安康博物馆开工建设，汉阴凤堰古梯田移民生态博物馆建成开放，被列为陕西文物工作十件大事之一。文庙二期工程竣工投用。通过剧作签约、班社扶持、资助奖励等措施振兴汉剧。建成100家数字农家书屋，完成15万户广播电视“户户通”工程。精神文明创建和群众文体活动丰富多彩。实施体育惠民工程137个。我市运动员夏江波在第14届伦敦残奥会上获得游泳双冠军，吴祺取得优异成绩。

　　（六）创新社会管理，和谐社会建设开创新局面。大力开展信访工作落实年活动，九个县巩固信访“三无”成果，实现赴省进京非正常上访“两下降”，全市信访总量连续三年下降。强化公安基层基础建设，不断完善社会治安防控体系，扎实做好社会敏感时段安保维稳工作，深入开展严打整治行动和打黑除恶专项斗争，社会治安动态管控能力提高，社会治安满意率达到95.9%。支持人民法院、人民检察院依法独立履行审判权、检察权，充分发挥惩治犯罪、促进和谐的职能作用。扎实开展万名执法人员规范执法承诺活动，强力推进省级依法行政示范市创建。深入开展“六五”普法，加强法律援助服务,设立仲裁委员会分支机构。积极应对处理网络热点舆情事件。深入开展安全生产打非治违活动，建立健全安全事故监管联动机制，推进高危行业标准化建设，整改安全隐患4588项，安全生产形势保持稳定。集中开展食品药品安全整治。科学制定落实防汛防滑预案，全面落实预警到户、提前转移、科学调度措施，最大限度减少汉江洪峰影响和山洪地质灾害损失，受到省防总通令嘉奖。高度重视做好国防动员、优抚安置、双拥共建、人民防空等工作，各条战线、各行各业在发展与和谐大局中都做出了积极贡献。各位代表，过去的一年，我们认真落实市委的决策部署，自觉接受市人大及其常委会的依法监督和市政协的民主监督。办理人大代表意见建议217件、政协委员提案291件，办结率100%。在全市政府系统力倡真抓实干，力戒慕图虚荣；力倡勤俭节约，力戒铺张浪费；力倡公开透明，力戒暗箱操作；力倡以身作则，力戒自由散漫；力倡深入群众，力戒高高在上，作风转变年活动成效显著。积极争取国家发改委、财政部、环保部、农业部、水利部、南水北调办、扶贫办等部委及省上厅局对安康发展的支持。出台实施加强政府投资项目和资金管理办法，深入开展工程建设领域突出问题专项治理。实行政府常务会议市民代表旁听制度。坚持重大决策广泛征求意见和风险评估制度。加快完善基层便民服务体系。制定实施推进重点工作落实的纪律规定和督查办法。查处破坏投资环境案件178件。政府执行力、公信力和工作透明度明显提高。

　　各位代表，我们在建设美好安康新征程上取得的成绩，是市委总揽全局、正确领导的结果，是市人大及其常委会依法监督、市政协民主监督和社会各方面大力支持的结果，是全市人民团结奋斗、拼搏进取的结果。在此，我代表市人民政府，向全体人大代表、政协委员，向奋战在各行各业、各条战线上的工人、农民、知识分子和干部职工，向各民主党派、工商联、人民团体、民族宗教、各界人士和离退休老干部，向驻安人民解放军、武警官兵、政法干警和复转军人，向所有关心支持安康建设的各界朋友，致以崇高的敬意和衷心的感谢！

　　在总结工作成绩的同时，我们也要清醒地看到，我市工业化程度较低，缺少大项目、大企业、大产业、大品牌带动，仍处在爬坡攻坚阶段；资源综合利用水平较低，节能减排降耗形势严峻，发展方式还没有实质性转变；对外开放水平较低，基础设施瓶颈尚未完全打破，投资环境亟待改善，承接产业转移的意识和能力不强；群众收入水平较低，新增就业岗位有限，增收致富渠道不宽，深山区群众生活还比较困难；一些地方、部门和干部服务发展的能力较低，服务意识不强、作风不实，吃拿卡要等现象还不同程度存在；还有财政收支矛盾突出、应对突发事件的能力亟待提高等问题。对这些问题，我们必须采取有力措施切实加以解决。

　　二、2013年工作的总体要求和目标

　　新的一年，是我们深入贯彻落实党的十八大精神、加快美好安康建设的关键一年。面临着诸多有利条件和难得机遇，一是党的十八大把生态文明建设纳入“五位一体”总布局，我省把“生态美”作为西部强省建设的重要目标。随着南水北调中线工程供水在即和国家主体功能区规划的深入实施，国家必将进一步完善生态资源补偿和生态功能区建设的扶持政策，更加有利于我市发挥生态优势、加快循环发展。二是国家优先推进西部大开发，将有力促进东中部产业向西部转移，关天、成渝、江汉、中原经济区转型发展，为我市加强区域合作、提高开放型经济水平创造了更加有利的条件。三是今年我市人均生产总值将超过3000美元，步入工业化、城镇化快速发展阶段，正赶上国家大力推进工业化、城镇化、信息化、农业现代化建设，“四化”同步政策效应的发挥，陕南移民搬迁工程的加快实施，必将强力推进我市城镇化大幅提速。四是今年将进入中省“十二五”规划、秦巴连片特困区扶贫攻坚规划集中实施阶段，省委、省政府进一步加大陕南循环发展扶持力度，我市又是全省唯一的全国发展改革试点城市，纳入省级战略层面的安康高新区、恒口示范区、月河川道现代农业基地和新型材料工业基地，将更加有利于争取中省的政策和项目支持。我们一定要抓住机遇，乘势而为，奋力开创美好安康建设新局面。

　　2013年政府工作的总体要求是：高举中国特色社会主义伟大旗帜，坚持以邓小平理论、“三个代表”重要思想、科学发展观为指导，深入贯彻落实党的十八大、省十二次党代会和市三次党代会精神，突出循环发展、富民强市主题，紧扣转变经济发展方式主线，构建循环产业体系，推进城乡统筹发展，改善基础设施条件，加快生态文明建设，不断深化改革开放，着力保障改善民生，促进文化发展繁荣，确保经济持续健康发展和社会和谐稳定，为全面建设美好安康，全面建成小康社会打下坚实基础。

　　按照市委三届三次全会的安排部署，今年经济社会主要发展指标建议为：生产总值增长13.5%；全社会固定资产投资增长25%；财政总收入增长13%，其中地方一般预算收入增长16%；社会消费品零售总额增长16%；城镇居民人均可支配收入增长14%，农民人均纯收入增长15%；城镇登记失业率控制在4%以内；人口自然增长率控制在4.5‰以内；完成省定主要污染物减排和节能降耗任务，汉江出境断面水质保持国家Ⅱ类标准。

　　三、经济社会发展的主要任务

　　（一）着力构建循环产业体系。做大新型工业。加快传统工业和骨干企业改造升级步伐。新型材料工业要突出抓好天沐金属镍、华银钒氮合金、宝钢铌钽采矿、陕煤石煤综合利用、旬阳铟锗回收、汉滨硫化钡、旬阳金矿、平利重晶石和纳米涂料等重大项目建设。大力支持中国五矿、宝钢集团、陕西煤化、陕西有色、紫金矿业等大型企业，控股、收购、兼并、参股矿产资源开发，延长产业链条，综合利用资源，提升整体水平。清洁能源工业要重点加快安康火电厂和旬阳、白河水电站建设，推进国电光伏、白河光伏电源项目，促进重点水电企业提质增效，支持生物能源和风能开发。富硒食品工业要着力打造“中国硒谷”品牌，做大做强矿泉水、茶饮、肉制品、粮油、魔芋食品等龙头企业，实施旬阳烟厂30万大箱技改项目，提高市场竞争力和占有率。安康丝绸工业要大力促进宝业丝绸、巴山丝绢、石泉天成、永丰特种纱等企业技改扩能，引进纺织、印染、服装、家纺、工艺品等精深加工企业，增强抗御市场风险的能力。生物医药工业要积极支持企业技术改造、新产品研发和原料基地建设。推动战略性新兴产业健康发展，高度重视和大力支持装备制造、电子信息、现代物流产业的发展。通过政府采购、财政奖补和市场开拓，促进汽车装配企业技改扩能，确保三一重工机械再制造项目竣工投产，加强与西安、重庆、十堰大型汽车生产企业合作，力争把我市建设成为陕渝鄂汽车产业配套协作区和配件生产加工基地。利用与关天、成渝、江汉国家信息制造研发基地的地缘优势，启动建设月河川道电子信息产业园区，认真研究、积极争取建设与西安三星电子项目的配套产业，引进培育一批高成长性的电子信息企业。加快现代物流园区和市场建设，培育区域性物流中心和商品集散地。加紧谋划和包装一批新项目、大项目，尤其是工业翻身项目。

　　做优现代农业。以“千元增收提增速、百园建设上水平”为主线，按照区域布局、板块推进的要求，加强对一县一业农业主导产业的规划和指导，确保有规模、有品牌、有效益。引导工商资本下乡、务工能人返乡创办农业企业，支持龙头企业发展产业联盟，培育市级以上龙头企业50户，发展农民专业合作社800个。创新土地流转方式，促进规模化、集约化经营。健全特色农产品市场体系，发展农产品物流配送企业。继续推进猪沼菜循环农业建设，全年粮油总产稳定在90万吨以上，生猪出栏310万头，新增设施蔬菜2万亩。大力发展山林经济，主攻茶叶、核桃、林下种养三大主导，稳定和提高蚕桑、烤烟、木本油料、木本药材、食用菌等特色优势产业。大力发展深加工龙头企业，加快种养型向加工销售型转变。继续扩大57个山林经济园区规模，启动建设50个山林经济园区。加快水资源综合开发利用，大力发展涉水产业。加快实施高新区康师傅饮品项目，支持陕煤富硒矿泉水做大做强，引进1到2家国内知名饮用水企业在安康建设分厂。加快推进生态渔业发展，打造安康富硒水产品牌，推动水资源大市向涉水产业强市迈进。建成我市与中国农科院共建的富硒产品研发中心，完成国家级实验站和博士后流动站组建，加快富硒农产品系列开发。健全完善农业科技服务体系，支持科研院所参与现代农业园区建设。加大新型职业农民培育力度，积极培育新型农业经营体系。加快秦巴山区安康市域发展与扶贫攻坚规划的实施，全面启动教育扶贫试点市建设，抓好60个片区172个贫困村连片开发整村推进项目，加大412个新农村示范村建设力度，实施10个国家级产业化扶持项目，支持20家扶贫龙头企业发展壮大，建设180个扶贫互助资金项目村，全年实现10万人脱贫。

　　做强生态旅游。坚持以山水为基础、人文为灵魂、项目为支撑、市场为核心，促进生态旅游与汉水文化、特色板块与4A景区、安康旅游与国内知名景区融合，推进门票经济向综合产业、单体景点向集群发展、观光旅游向休闲度假转型，加快形成汉水风情休闲、巴山生态度假、秦岭森林体验、田园风光感受等特色精品线路，建设南水北调水源地生态文化旅游产业示范区和国内一流生态旅游目的地。大力实施“一山一湖一城”核心景区提升工程，完成大南宫山主景区及配套开发建设；以瀛湖体制调整为契机，突出资源整合和招商引资，引进大企业开发建设大瀛湖景区；精心打造一江两岸核心区，加快高星级酒店及配套设施建设，带动服务业发展，增强中心城市旅游集散功能。建成天书峡、神河源景区，扩建燕翔洞和中坝大峡谷，开发太极城、鬼谷岭、红石河、凤凰山，推进香溪洞、朝阳沟、冰晶顶、深山老林等项目建设。支持石泉创建全省第二批旅游示范县。启动第二批旅游示范村镇和示范户建设。以汉江龙舟节为主体，办好节庆促销活动。全年接待游客2000万人次。

　　做活园区经济。高新区要以创建国家级高新技术产业开发区为目标，突出产业聚集区、现代城市新区、统筹城乡示范区的定位，全面推进陕煤化、华银集团、湖北金龙、鑫州矿业、渝企工业园等总部经济和重点产业项目，建成数字创业中心、三所学校、三个生态公园和一批基础配套设施。确保生产总值增长30%以上。突出县域工业集中区建设，创新产业组织形式，加快循环产业园区生产要素、产业项目、资源聚集和基础配套，每个园区重点打造2至3个循环经济链条，集中培育1个产值过50亿元、4个过20亿元的县域工业集中区。扶持劳动密集型产业发展，引进50户企业、培训1万名技术工人、带动5万人就业。加快全市254个现代农业园区建设，培育和建设农产品物流园、加工园、休闲观光园，建成5个产值5000万元以上的农业园区。（二）全力加快城镇化步伐。高度重视产业兴城。科学合理规划和布局，推行经济社会发展规划、城乡规划、土地规划“三规合一”，形成聚集人口效应，使城镇建设与产业布局相衔接。坚持城乡产业统筹，切实增强园区综合承载能力，优先发展劳动密集型产业和三产服务业，扩展就业空间，促进进城农民市民化，奠定城镇化发展的坚实基础。

　　加快发展中心城市。加大城市北迁、江南旧城改造、城市绿化美化力度。加快已确定的13个单位北迁项目建设。建成滨江大道三期、南环东路、高新大道一期等一批城市道路，开工实施新城文化街区、人防地下商业街、江北自来水厂、滨江一号地等重点项目，加快推进管网配套、强电入地、停车场、市场和环卫等市政设施建设。突出抓好大绿围城、公园绿地、道路绿化、庭院绿化、城市出入口绿化等绿化美化工程，让绿色真正成为美好安康的象征。推行“大城管”管理模式，促进“数字化”城市建设，实施城市精细化管理。全面实现中心城市国家园林城市创建目标，通过国家卫生城市国家技术评估。启动省级和国家级环保模范城市创建工作。坚决维护规划的严肃性，依法严厉打击违法用地、违法建房行为。

　　科学建设现代城镇。按照做美城市、做强县城、做大集镇、做好社区的要求和建设集约、生态、服务型城镇的思路，大力促进城乡一体化发展。注重彰显城镇个性，推动县城和30个市级重点镇扩容升级，建设特色集镇和60个新型社区，不断完善市域城镇体系。强力推进月河川道城镇带建设，加快恒口示范区镇级市建设。支持县城提升建设水平，引领县域经济社会发展。结合避灾扶贫搬迁、保障性住房、农民进城、产业园区建设，培育一批工业强镇、旅游大镇、商贸重镇、文化名镇。完善社区管理服务机制，加强社区服务设施建设，提高公共管理和服务能力。按照四级联创要求，深入推进县城双创工作，宁陕、汉阴创建为国家卫生县城，汉阴、石泉创建为国家园林县城。

　　　（三）大力推进生态文明建设。牢固树立生态文明理念。良好生态环境是安康人民的福祉所在、财富所在、美好未来所在。要深刻领会党的十八大“五位一体”总布局的精神实质，全面贯彻市委、市政府加快生态安康建设的实施意见，把生态文明建设融入安康经济社会发展的各方面和全过程。强化生态协同产业发展，让城乡因生态而美，让群众因生态而富。努力在“生态美”上领跑全省，在生态建设上争做全国示范，永葆安康山清水秀、天蓝地绿。

　　加快实施造林绿化工程。建立全民植树造林常态化机制，认真实施山地森林化规划。大力实施丹治二期、汉江综合整治、天然林保护、长江防护林等重点生态工程。突出中心城区大绿环、月河川道绿化带、公园道路绿化群建设，集中力量抓好人口密集、生态脆弱、缺林少绿区域的绿化及墓地绿化。加快月河等流域生态系统修复。完成造林绿化投资3亿元，造林绿化50万亩，义务植树700万株。

　　强力推进节能减排降耗。把节能减排降耗作为生态建设的硬任务，促进生产、流通、消费过程减量化、再利用、资源化。建立能源消费评估机制。全年实施12个重点节能减排降耗工程，节能12万吨标煤。启动10户企业清洁生产示范项目，建成20个畜禽养殖污染治理工程。城市污水集中处理率82%以上，城市生活垃圾无害化处理率90%以上，启动5个重点镇污水管网项目建设。实施瀛湖生态环境保护工程。加大水泥企业烟气脱硝治理和黄标车淘汰力度，推广清洁养殖、清洁种植、清洁消费。

　　严格实行生态保护制度。全面落实节约资源和保护环境的基本国策。认真实施国家主体功能区规划，有效控制限制开发区开发强度。加强国土空间开发保护，推进土地综合整治，保持耕地占补平衡，提高土地利用效益。强化环境监管和责任追究，环境影响评价和“三同时”执行率达到100%。大力整治水、大气、粉尘、噪声、土壤等污染问题，强化重金属污染防治，严控二氧化硫、危险废弃物排放，扎实推进矿山复绿，严禁破坏山体、河流生态，严肃查处环境违法行为。汉阴、白河创建为省级生态县。城市空气优良天数稳定在350天以上。城镇饮用水源地水质达标率100%。

　　（四）扎实推动重点项目建设。突出抓好十大项目建设。十大项目关乎安康今后的发展基础，一定要集中精力，攻坚克难，加快推进。开工建设西坝旧城改造、安康机场试验段、中心城市北环线试验段、汉江四桥、旬阳县污水处理厂项目，加快实施中心城区道路绿化、汉江干流县城防洪、安康火电厂、金属镍产业园项目，积极推进东坝综合开发项目前期工作，当年完成投资32亿元。扎实抓好100个基础设施、产业建设、民生工程、生态环保市级重点项目，以重点项目建设扩投资、保增长、增后劲。

　　加快交通基础设施建设。全线开工安平高速公路，建成西康铁路二线和紫阳两座汉江大桥，加快建设西成铁路客运专线，启动实施阳安铁路二线。完成平镇路大中修，加快白河县城国道过境段改造，开工316国道安康城区过境段、汉阴段一级公路，做好安康至旬阳、旬阳至小河二级公路开工准备。全面实施秦巴连片特困地区交通扶贫项目，实施石泉至紫阳至岚皋新增国道项目和12条县区经济主干线建设。加快建设铁路、高速公路、地方道路互通互联的配套路网，尽快恢复高速公路建设损坏的地方道路。全面完成万公里通村示范路，新建300公里县镇示范路、3000公里连户路。加快安康高客站、城北和紫阳、岚皋客运站建设。推进安康至白河高等级航道和中心港项目。积极争取平镇高速公路挤进中省项目计划。

　　加强重点水利工程建设。全面加快汉江综合整治，重点推进东坝和6县区16个镇的防洪保安工程。加快实施引汉济渭、洞河水库等骨干水源项目，做好月河补水工程前期工作，推进6县城自备水源项目建设。解决22万人的安全饮水问题。实施28条小流域丹治二期、10条中小河流治理工程，治理水土流失面积1000平方公里。完成15座病险水库除险加固。继续实施“五小”工程，新修基本农田5万亩，新增节水灌溉2万亩。

推进公共基础设施建设。着力解决江北新区、产业园区、新型社区和重点企业用电问题，开工建设旬阳330千伏变电站，加快实施东郊110千伏输变电项目，加大城乡电网改造力度。完成中心城区和汉阴、石泉县天然气配套管网工程。改革公交管理体制，优先发展城市公共交通，加快编制中心城区公交发展规划，优化城区公交线路，增加公共交通运力。

　　（五）努力提升改革开放水平。深化重点领域改革。深入推进全国发展改革试点城市工作，靠改革推动经济持续发展。按照职责明确、权责统一、运行高效的原则，加快推进市区管理体制改革。支持高新区、恒口示范区、瀛湖生态旅游区创新体制机制。积极推进统筹城乡综合配套改革。实施城市污水和垃圾处理收费改革，确保污水和垃圾处理设施正常运行。按照中省安排推进财税体制改革，不断完善公共财政体系。加快林权制度配套改革。持续推进县级公立医院改革，制定市级医疗机构发展规划，实行市级医疗单位药品集中招标和最高限价，逐步建立现代医院管理制度。完成事业单位分类改革清理规范阶段任务。

　　加大招商引资力度。把招商引资放在更加突出的位置，创新招商方式，拓展招商领域，提高招商实效。在招商项目策划上突出循环产业链条和产业集群项目，根据产业工业化流程接环补链，推动产业由低端的原料生产向中高端产品生产延伸。在招商项目布局上统筹考虑，实行重大产业市县区联建共建，科学定位县域和产业园区主导产业发展重点，避免在同一区域内的产业同质。主动承接长三角、珠三角、环渤海等发达地区的产业、资本和技术转移，加强与关天、成渝、江汉等周边经济圈产业的对接融合，密切与北京石景山区、江苏常州市和中西部经济协作区的合作。有计划、有目的地开展招商活动，引进一批知名企业落户安康。全年招商到位资金210亿元。要继续优化投资环境，大力开展“信用政府、诚信安康”建设。凡是出台的优惠扶持政策要坚决落实到位，凡是招商合同承诺事项要坚决兑现到位，凡是破坏投资环境的案件要坚决处理到位，真正让投资者放心安心舒心。

　　切实改善金融服务。充分发挥金融机构融资主渠道作用，利用融资平台撬动社会资本。规范发展小额贷款公司和担保机构，多渠道扩大融资规模。安康发展投资集团实现首期10亿元发债，提早做好二期发债准备工作。支持高新产业发展集团公司提升发债融资能力。提高市财信担保公司担保能力。新增融资性担保公司和小额贷款公司各2家。密切与金融机构的合作，积极推动股份制商业银行在安康设立分支机构和村镇银行，改建2家有条件的农村信用联社为农村商业银行。促进银企对接，定期通报产业政策、推介重点项目，建立项目洽谈金融机构提前介入机制。实施市级财政专项资金存放商业银行管理制度。支持金融机构转变赢利模式，创新金融产品，降低贷款门槛，简化审批环节，提高综合效益。

　　（六）切实加强民生为重点的社会建设。突出抓好教育第一民生。旬阳、石泉县实现国家级义务教育均衡发展县目标，宁陕县命名为省级教育强县，白河县创建为省级“双高双普”县，镇坪、汉阴、岚皋县全面启动“双高双普”工作。加快高新中学、白河中学和平利二中建设，扩大中心城市和县城高中办学规模，高中阶段入学率提高到88%。支持平利、白河县实施高中免费教育。新改扩建41所县城标准化幼儿园和镇办中心幼儿园，学前教育三年入园率提高到78%。加快实施义务教育薄弱学校改造计划、初中校舍改造工程、学前教育推进工程。深化和完善城乡义务教育经费保障机制改革等政策，全面实施义务教育营养改善计划，健全农民工子女上学、留守儿童教育管护、家庭困难学生资助体系。完善安康学院江北校区功能，加快安康职业技术学院北迁进度。发展现代职业教育，规范发展民办教育和继续教育，更加重视特殊教育。强化校园安全管理。突出抓好教育质量，深入开展“名校、名师、名校长”争创活动，在全社会形成崇文、尊师、重教、兴学的浓厚氛围。

　　大力实施就业优先战略。千方百计扩大就业规模，扶持发展就业容量大的劳动密集型产业、三产服务业、现代农业园区和小微型企业。推广白河县“移民搬迁进社区、土地流转建园区、农民就地变工人”的做法，形成搬迁、进城、就业的良性循环。强化公共就业援助服务，引导高校毕业生到农村基层、城镇社区和企业就业，统筹推进农村劳动力、城镇困难人员、复员退伍军人和失地农民就业。新增城镇就业15300人，大学生就业3500人，免费技能培训2.3万人，发放小额担保贷款5亿元。全面实行劳动合同和用工备案制度，推行企业工资集体协商制度，依法维护劳动者合法权益，构建和谐劳动关系。

　　加快完善社会保障体系。继续抓好避灾扶贫搬迁工程，推进跨区域集中安置，重视危居困难群众搬迁，加快产业就业创业配套建设，确保“搬得出、稳得住、能致富”。较高质量地完成2.2万户8.36万人的搬迁任务。新开工保障性住房3.36万套，其中廉租房、公租房各5000套，优先解决低收入群众的住房困难。提高城乡居民社会保险覆盖水平，养老、医疗参合参保率95%以上，在月河川道重点镇开展城乡居民社会保障制度一体化并轨试点，鼓励有条件的县区先行先试。扎实做好城乡低保、五保供养、医疗救助、临时救助等社会救助工作，中心城区老年公寓和社会福利院建成投用，积极发展民办养老机构，五保集中供养率提高到60%。

　　提高公共医疗服务水平。健全重大疾病市级统筹保障机制，新农合住院费用平均报销比例提高到75%以上。加快县医院、中医院、乡镇卫生院等29个项目建设，规范11类公共卫生服务项目管理，健全医疗卫生服务体系，提高应对突发公共卫生事件和重大疾病防控能力。促进中医药产业和民营医疗机构发展。加强医德医风建设，依法打击冲击打砸医疗机构行为，构建和谐医患关系。巩固基本药物制度，加大食品药品安全整治和监管力度。认真落实计划生育基本国策，建立以县为主统筹解决人口问题机制，提高计划生育综合执法水平，完善计生利益导向政策体系，促进人口长期均衡发展。

　　推进文化事业发展繁荣。全面落实市委、市政府关于人文安康建设的意见。加快完善公共文化服务体系，启动创建全国公共文化示范项目，抓好安康博物馆、图书馆、汉调二黄园、西城阁、重点镇文体中心等项目建设，完成34万户广播电视“户户通”工程。加强文化遗产保护，深入实施振兴汉剧工程，重点抓好人才培养、班社扶持和精品剧目打造，抓好凤堰古梯田、北五省会馆等一批重点文物的保护与开发，扩大紫阳民歌、龙舟文化影响力。制定实施安康文化产业中长期发展规划,培养文化领军人物，打造具有安康特色的文化品牌。加强文化市场管理，切实规范市场秩序。以宣传党的十八大精神为主题，深入开展文化、科技、卫生下乡等活动。加快实施农民健身和县级全民健身中心工程，办好安康市第十五届运动会。继续解决城区三难问题。中心城区住院难、上学难、行车难仍然是群众关注的热点问题，必须逐年消化解决。加快市儿童医院、妇幼保健院、精神病专科医院和全科医师培训基地项目建设。建立有效的市县医院与基层医疗机构转诊协作机制，整合市级卫生资源，提高基层医院服务能力。今明两年安康城区再增加1000张病床；加快城区学校布局调整，健全师资力量动态调配机制，强化强校带弱校、对口支援帮扶措施，秋季入学前完成汉滨高中、汉滨初中、石堤小学、市一小等8个改扩建项目，进一步缓解“大班额”压力；加快城市交通基础设施建设，优化重点路段、重点时段的交通组织，逐步完善交通智能化和管制系统，提高市民文明安全出行意识。努力让老百姓不再因为上学、住院、出行的问题而烦恼。

　　加强和创新社会管理。认真做好重大决策、重大项目、重点改革实施前的社会稳定风险评估。强化基层组织服务功能，创新新型社区管理体制。完善信访工作机制，健全“三位一体”调解体系，坚持领导干部接访、约访、下访、回访制度，继续抓好“三无”县区创建，依法规范信访秩序，畅通群众诉求表达、利益协调、权益保障渠道，及时排查矛盾隐患，把问题解决在基层。深化平安安康建设，健全完善治安防控体系，深入开展严打专项整治，提高对违法犯罪活动打击、防范、控制能力，不断增强群众安全感。强化特殊人群管理和服务。扎实抓好“六五”普法，提高全民学法尊法守法意识。依法加强虚拟社会管理，正确引导网络舆论，及时妥善处理舆论事件。大力弘扬“安康精神”，增强群众社会责任感，培育良好的社会心态。强化安全发展意识，加快公共安全体系建设，全面落实安全生产责任制，深化打非治违专项行动，加大安全隐患排查整治力度，推进企业安全标准化建设，严防重特大安全事故发生。以7.31安康特大洪灾30周年纪念为契机，深入开展防汛减灾警示教育活动。完善汛期防抢撤预案，提高预测预警、灾害防范、应急处置能力，切实保障人民生命财产安全。

　　坚持男女平等基本国策，维护妇女儿童合法权益。做好国防动员、双拥共建、优抚安置和人民防空工作。切实加强老龄服务，支持慈善、红十字事业发展。健全残疾人社会保障制度。着力解决少数民族群众生产生活困难。依法管理宗教事务。认真做好外事、侨务、对台、史志、档案工作，支持工会、共青团、妇联等人民团体在美好安康建设中发挥更大作用。

　　四、不断强化政府自身建设

　　（一）坚持依法行政，促进权力公开透明运行。自觉把依法行政贯穿于政府决策、执行、监督的全过程，严格按照法定权限和程序行使权力。完善政府科学民主决策机制，把公众参与、风险评估、合法性审查和集体讨论决定，作为重大行政决策的必经程序，确保权限合法、实体合法、程序合法。以法律规范政府行为，靠法律调处社会矛盾，用法律维护市场秩序。深入推进依法行政示范市县创建，实行行政处罚自由裁量基准制度，整合组建中心城区综合行政执法机构，切实解决执法缺位、错位问题，做到规范、公正、文明执法。建立行政调解指挥中心，发挥行政裁决、行政复议、行政调解作用，引导群众通过法定途径反映诉求、解决纠纷、维护权益。主动接受市人大及其常委会依法监督、市政协民主监督和社会舆论监督，让行政权力在阳光下运行。

　　（二）狠抓工作落实，营造真抓实干浓厚氛围。为政不在言多，贵在实干。政府的职责就是抓落实。要把精力集中到加快发展上，把心思用在改善民生上，把功夫下到狠抓落实上。健全抓落实的工作机制，把重点工作、重要任务、发展目标分解到部门、具体到项目、落实到岗位、量化到个人，制定强有力的组织措施、考核措施、激励措施，完善人人负责、环环相扣、科学合理、行之有效的责任制。坚决纠正推诿扯皮、回避矛盾、消极应付的顽疾。要敢于负责、敢于担当、敢于较真、敢于碰硬。坚持凭实绩看干部，以成效论英雄，让那些肯干事、能成事的干部有舞台、有作为，形成比学赶超、奋发向上、脚踏实地、真抓实干的生动局面。

　　（三）切实改进作风，密切与群众的血肉联系。全面对照中央政治局关于改进工作作风、密切联系群众的八项规定和省委实施意见，查找存在问题，认真进行整改。加强各级政府和领导干部作风建设，坚决反对走过场、搞形式、图虚名，大兴密切联系群众、求真务实、艰苦奋斗之风。要设身处地为群众着想，把群众当亲人，把群众事当家里事，用真情做好工作，用行动赢得信任。要厉行勤俭节约，坚持勤俭办一切事情，把每一分钱都用在发展和民生上，严格控制行政开支，推进部门“三公”经费公开，精简会议活动，整治文风会风。按照干部清正、政府清廉、政治清明的要求，兴清正之风，倡廉洁之行。积极推进惩防体系建设，严肃查处违法违纪案件，把廉政建设的各项任务融入到政府工作之中，建设为民务实清廉的人民政府。

各位代表，安康发展的目标激励着我们，身上肩负的责任鞭策着我们，只要我们咬定青山不放松，团结奋斗不懈怠，就一定能够在加快循环发展、建设美好安康的伟大征程中迈出更加坚实的步伐。让我们高举中国特色社会主义伟大旗帜，全面贯彻落实党的十八大战略部署，在市委的坚强领导下，紧紧依靠全市人民，夙夜在公，扎实苦干，开拓创新，以优异的成绩向全市人民交一份合格的答卷。
